# Supplementary material for: Traveler’s knowledge, attitude, and practice about travel health insurance: A community-based questionnaire study
Source: PLoS One. 2023 Feb 9;18(2):e0281199. doi: 10.1371/journal.pone.0281199 (PMC9910690; doi:10.1371/journal.pone.0281199)
Supplement: Attachment — Submitted filename: Reviewer attachment.pdf [file pone.0281199.s003.pdf]

I suggest you add the study's design in the title and in the abstract.

In the abstract substitute:

Accident → accidents

visitors of → visitors to the

willingness → the willingness

the international → international

In the introduction substitute:

Increased → increasing

Tourisms → tourism

Responsibilities → responsibility

Noncompliant → non-compliant

1980s' → 1980s

Shown → showed / has shown

Categories → the categories

About → of

Risk-coverage → risk coverage

a travel → travel

77 - 80 – you should clarify this sentence. First you say it is the health providers responsibility and then you say travellers did not follow the suggestions. Maybe the responsibility is shared?

93 - 95 – How the first part of the sentence justifies the second part?

In general, the introduction is not very clear and effective in demonstrating the importance of health insurance for travel and have several writing issues. I recommend that the text be proofread by a native English speaker.

111 – 114 – You should clarify this paragraph. It is not clear what is the study design, the instrument and the participants.

117 – 119 – This phrase does not belong in the subjects' section.

In the subject' section you should add from where and how the participants were selected.

Results

In the results' section consider to include the number next to the percentage and also de p value next to the result presented.

The results presented in the tables should be better described in the text.

169 – selection → selections

175 – illness → illnesses

176 – self-guide → self-guided

187 – 189 – In this sentence what means "top three ones"?. I suggest: Travellers when asked about the most important insurances or to rate the insurances, 92% answered..

Discussion

216 – illness → illnesses

220 – from → of

222 – for → in

228-229 – I suggest you write it this way: The low percentage of travellers willing to seek pre-travel advice described in the literature contrasts with the percentage (exact percentage?) of those motivated to buy travel health insurance in this study.

239 – 240 – You should clarify this sentence. According to what you described in 238-239, this sentence should be the cost of travel health insurance for the traveller or you are describing how much it cost without insurance?.

241 - 243 – I suggest: Overseas sickness coverage and medical evacuation are crucial while traveling during the Covid-19 pandemic since local healthcare capacity can be overwhelmed.

244 – remove "Besides" and "also"

245 – about → of

249 – of → to

255 – toward → of

256 – remove "is"

257 – one third → one-third

267 - 60% general → 60% of general; give advice → advise; insurances → insurance

269 – the travelers → travelers; insurances → insurance

271 – insurances → insurance; a new → new

272 – airline → airlines

Conclusion

The conclusion should include something about knowledge related to infections, vaccines, and illnesses while traveling.

289 – 291 I suggest: between a third and half of the travelers understand the content of health insurance, almost all recognize its importance, and more than two-thirds were willing to buy a health insurance when traveling.

293 – 294 I suggest: Therefore, health professionals should be encouraged to acquire health insurance knowledge and transmitted it on during pre-travel consultation..

295 – consultation → consultations

Figure 1 must be redone, it is not possible to see due to lack of quality.
