# Supplementary material for: Traveler’s knowledge, attitude, and practice about travel health insurance: A community-based questionnaire study
Source: PLoS One. 2023 Feb 9;18(2):e0281199. doi: 10.1371/journal.pone.0281199 (PMC9910690; doi:10.1371/journal.pone.0281199)
Supplement: Attachment — Submitted filename: Response to Reviewers_20220802.docx [file pone.0281199.s002.docx]

**Replay to Edits**

We've checked your submission and before we can proceed, we need you to address the following issues:

1. Please provide additional details regarding participant consent. In the Methods section, please ensure that you have specified (1) whether consent was informed and (2) what type you obtained (for instance, written or verbal). If your study included minors, state whether you obtained consent from parents or guardians. If the need for consent was waived by the ethics committee, please include this information.

**Response:** Thanks for your recommendation. We stated that the purpose of this study was to understand travelers’ knowledge, attitudes, and practice at the beginning of the questionnaire. And all respondents gave verbal consent before they fulfilled the questionnaire. We have also shown “The study was approved by the Institutional Review Board at National Taiwan University Hospital in Taiwan (201902070W) before the study was conducted.” on page 7. The questionnaire was administered anonymously in public. We cannot recognize the personal identification after completion of the questionnaire. The Institutional Review Board at National Taiwan University Hospital in Taiwan agreed that the study could be conducted with oral informed consent.

**Reply to Editor**

When submitting your revision, we need you to address these additional requirements.
1. Please ensure that your manuscript meets PLOS ONE's style requirements, including those for file naming. The PLOS ONE style templates can be found at
https://journals.plos.org/plosone/s/file?id=wjVg/PLOSOne_formatting_sample_main_body.pdf and
<https://journals.plos.org/plosone/s/file?id=ba62/PLOSOne_formatting_sample_title_authors_affiliations.pdf>.

**Response:** Thanks for your recommendation. We’ve checked and amended our manuscript to meet PLOS ONE's style requirements.

2. Thank you for stating the following financial disclosure:
“This study has been partially sponsored by the Centers for Disease Control, Taiwan (JK108026).”Please state what role the funders took in the study.  If the funders had no role, please state: "The funders had no role in study design, data collection and analysis, decision to publish, or preparation of the manuscript." If this statement is not correct you must amend it as needed. Please include this amended Role of Funder statement in your cover letter; we will change the online submission form on your behalf.

**Response:** Thanks for your recommendation. We’ve added "The funders had no role in study design, data collection and analysis, decision to publish, or preparation of the manuscript." in cover letter.

3. In your Data Availability statement, you have not specified where the minimal data set underlying the results described in your manuscript can be found. PLOS defines a study's minimal data set as the underlying data used to reach the conclusions drawn in the manuscript and any additional data required to replicate the reported study findings in their entirety. All PLOS journals require that the minimal data set be made fully available. For more information about our data policy, please see http://journals.plos.org/plosone/s/data-availability.

Upon re-submitting your revised manuscript, please upload your study’s minimal underlying data set as either Supporting Information files or to a stable, public repository and include the relevant URLs, DOIs, or accession numbers within your revised cover letter. For a list of acceptable repositories, please see http://journals.plos.org/plosone/s/data-availability#loc-recommended-repositories. Any potentially identifying patient information must be fully anonymized.

Important: If there are ethical or legal restrictions to sharing your data publicly, please explain these restrictions in detail. Please see our guidelines for more information on what we consider unacceptable restrictions to publicly sharing data: http://journals.plos.org/plosone/s/data-availability#loc-unacceptable-data-access-restrictions. Note that it is not acceptable for the authors to be the sole named individuals responsible for ensuring data access. We will update your Data Availability statement to reflect the information you provide in your cover letter.

**Response:** Thanks for the instructions. We’ve uploaded minimal underlying data set as Supporting Information files, S1 File Minimal data set.

Additional Editor Comments:
We noticed that your manuscript title and abstract specifically mentions COVID-19. However, the survey was conducted in March 2019, just at the beginning of the global COVID-19 pandemic, and we assessed that the article was written to represent a much broader subject of international travel unrelated to COVID-19. We would therefore suggest removing the mention of COVID-19 from the title and abstract.

**Response:** We appreciate your comment. We’d like to adjust the title to **“Traveler’s Knowledge, Attitude, and Practice about Travel Health Insurance”**. We also removed the mention of COVID-19 from the abstract and keywords.

**Reply to Reviewer**

**Reviewer #1**

Reviewer #1: A few things to consider in revisions:
1. This study is a knowledge, attitudes, and awareness study, but I am curious as to why "practices" was not included? To me, awareness is the same as knowledge, so I think this important component was left out of the questionnaire.

**Response:** We appreciate your opinion. Actually, we included the travelers’ willingness toward visiting travel medicine clinics and buying travel health insurance, so we think that the revised title “**Traveler’s Knowledge, Attitude, and Practice about Travel Health Insurance**” is more appropriate.

2. I am not sure what is meant by "important travel insurances." Typically travel insurance has multiple facets, and one needs to look to see what coverage is.
**Response:** Thanks for your comments. We feel the same way, the importance of the travel health insurance depends on the coverage and the demand of the traveler. Because we want to clarify the attitude and the willingness of the travelers, we asked the travelers how do they think about different kind of insurance and will they buy that. The result was according to their reply.

3. Remove the study from the 1980s from the intro. It is old and not relevant.

**Response:** Thanks for your suggestion. We’ve removed the reference.

4. International SOS is not mentioned at all in the manuscript. As the largest provider of insurance and evacuation services globally they should be mentioned. They have also published findings they do not seem to be included.

**Response:** Thanks for your suggestion. We agreed and used their statistics to show the need of 24-hour emergency assistance is quit a lot, so we added “**According to the statistics of International SOS Group of Companies, they received 4 million assistance calls until 2022 [18]. The demand of 24-hour emergency assistance is quite a lot.**” in discussion section, Line 226-227, Page 17.

5. Were respondents consented? If yes or no, this should be mentioned in the methods.
**Response:** Thanks for your reminder. Yes. All respondents consented. We added that” **All respondents gave verbal consent before they fulfilled the questionnaire.”** in the Design paragraph, Line 109-110, Page 7.

6. One third of people had a chronic illness, which likely greatly impacted their responses. This should be discussed thoroughly in the discussion.

**Response:** Thanks for your recommendation. There was no significant association between chronic illness and the willingness to buy travel health insurance, thus we did not expound it (Table 2). However, we found that the knowledge of travel-related infection, vaccine, and diseases was significantly associated with medical history (Table 3).

We stated “**In addition, travelers with older age, medical histories, and travel-associated illnesses were significantly associated with a higher knowledge of travel medicine.** **In other words, those travelers who have the willingness to buy travel health insurance may not have a good knowledge of travel medicine**.” in discussion section, Line 233-237, Page 17.

7. All tables should be in descending numerical order. (example: purpose of trip is not in any sort of format). Right now each variable is not in descending order.

**Response:** Thanks for your recommendation. We’ve revised all tables, Table 1, 2 and 3.

8. The discussion should start with a paragraph on what makes this analysis important and unique.

**Response**: We appreciate for your recommendation. We’ve now revised the first paragraph of Discussion section and focused on the unique of this study.” **In our main findings, we clearly demonstrated that the top three important travel health insurances were accidental death and disablement insurance (92%), accidental medical reimbursement (90.4%), and 24-hour emergency assistance (89%). Furthermore, we found that the education level, travel-associated illness, and special activities during travel were significant parameters to influence the willingness to buy various travel health insurances and willingness of pre-travel consultation.”** (Line 199-204, page 16)

.
9. I am unclear what this means in the context of the discussion: "An increasing trend for international travel has been observed." Remove.

**Response**: Thanks for your suggestion. We’ve removed it.

10. Conclusions should not have any data. They should reiterate the take home points without data. I also think the conclusions need to be strengthened. What is the public health and clinical importance of the findings?

**Response**: Thanks for your comments. We’ve revised the paragraphs of conclusion. Our study highlights the gap between travelers’ willingness to buy and to understand travel health insurance. Most travelers thought that healthcare workers are the most reliable source, so we should provide them more comprehensive advice, including travel health insurance.” **Most travelers would buy travel health insurance, such as accidental death and disablement insurance and accidental medical reimbursement; however, a disproportionate percentage of the respondents understood the content very well. Just buying insurance does not equal to being well-prepared.**” Line 261-264, Conclusions section, page 20.

11. Each paragraph of the discussion should be expanded on. Paragraphs should start with a strong introductory sentence, available evidence, and then followed by a strong closing.

**Response**: Thanks for your comments. We’ve revised every paragraph of discussion according to your suggestion. Discussion section, page 16-18.

12. I do not think that Figure 1 adds much. Remove.

**Response**: Thanks for your suggestion. We’ve removed it.
