# Supplementary material for: Traveler’s knowledge, attitude, and practice about travel health insurance: A community-based questionnaire study
Source: PLoS One. 2023 Feb 9;18(2):e0281199. doi: 10.1371/journal.pone.0281199 (PMC9910690; doi:10.1371/journal.pone.0281199)
Supplement: Attachment — Submitted filename: Response to Reviewers_202211107.docx [file pone.0281199.s004.docx]

**Reply to Reviewers**

**Reviewer #2:**

**1. I suggest you add the study’s design in the title and in the abstract.
Response:** Thanks for your recommendation. We have added the study’s design in the title, “**Traveler’s Knowledge, Attitude, and Practice about Travel Health Insurance: A Community-based Questionnaire Study**.” We also added the design in the abstract. “**Methods: This was a cross-sectional study. Anonymous structured questionnaires were distributed to 1000 visitors to the Taiwan International Travel Fair in May 2019**.”

**2. In the abstract substitute:**
Accident --> accidents
**Response:** We have revised the word. (LINE 34)

visitors of --> visitors to the
**Response:** We have revised the word. (LINE 40)

willingness --> the willingness
**Response:** We have revised the word. (LINE 45)

the international --> international
**Response:** We have revised the word. (LINE 36)
**In the introduction substitute:**
Increased --> increasing
**Response:** We have revised the word. (LINE 70)

Tourisms --> tourism
**Response:** We have revised the word. (LINE 72)

Responsibilities --> responsibility
**Response:** We have revised the word. (LINE 76)

Noncompliant --> non-compliant
**Response:** We have revised the word. (LINE 77)

1980s’ --> 1980s
**Response:** We’ve removed this reference according to another reviewer’s suggestion.

Shown --> showed / has shown
**Response:** We have revised the word. (LINE 87)

Categories --> the categories
**Response:** We’ve removed this sentence according to another reviewer’s suggestion.

About --> of
**Response:** Thanks for your recommendation. We’ve removed this sentence according to another reviewer’s suggestion.

Risk-coverage --> risk coverage
**Response:** We’ve removed this sentence according to another reviewer’s suggestion.

a travel --> travel
**Response:** We’ve revised the word. (LINE 94)
**3. 77 - 80 – you should clarify this sentence. First you say it is the health providers responsibility and then you say travelers did not follow the suggestions. Maybe the responsibility is shared?**
**Response:** We have revised the sentence to avoid the misunderstanding. (LINE 75-76)
**4. 93 - 95 – How the first part of the sentence justifies the second part?
Response:** Thanks for your question. Initially, we wanted to emphasize that although there were many kinds of travel health insurance, few studies investigated travelers’ knowledge, attitude, and practice. Thus, we conducted this study. To make it clearer, we removed the redundant sentences. (LINE 92-99)

**5. In general, the introduction is not very clear and effective in demonstrating the importance of health insurance for travel and have several writing issues. I recommend that the text be proofread by a native English speaker.
Response:** The introduction section is revised carefully. We have also sent it to the English editing by a native English speaker.

**6. 111 – 114 - You should clarify this paragraph. It is not clear what is the study design, the instrument and the participants.
Response:** We’ve re-organized the paragraph of Design and Subjects to make them clearer. (LINE 104-112)

**7. 117 – 119 – This phrase does not belong in the subjects’ section.**
In the subject’ section you should add from where and how the participants were selected.
**Response:** We’ve re-organized the paragraph of Design and Subjects to make them clearer. (LINE 104-112)

**8. Results
In the results’ section consider to include the number next to the percentage and also the p value next to the result presented.
The results presented in the tables should be better described in the text.
Response:** Thanks for your recommendation. We’ve included the number and the p value next to the result presented. (Results paragraph)

**9. Grammar correction**

169 – selection --> selections
**Response:** We have revised the word. (LINE 162)

175 – illness --> illnesses
**Response:** We have revised the word. (LINE 167)

176 – self-guide --> self-guided
**Response:** We have revised the word. (LINE 169)

**10. 187 – 189 – In this sentence what means “top three ones”? I suggest:** **Travelers when asked about the most important insurances or to rate the insurances, 92% answered.
Response:** We appreciate your recommendation. Because another reviewer’s suggestion, we’ve adjusted this main finding to our discussion session. And we have revised the grammar according to your recommendation. (LINE 204-207)

**11. Grammar correction**

**Discussion**
216 – illness --> illnesses
**Response:** We’ve removed this sentence according to another reviewer’s suggestion.

220 – from --> of
**Response:** We’ve removed this sentence according to another reviewer’s suggestion.

222 – for --> in
**Response:** We’ve removed this sentence according to another reviewer’s suggestion.

**12. 228-229 – I suggest you write it this way:** **The low percentage of travelers willing to seek pre-travel advice described in the literature contrasts with the percentage (percentage) of those motivated to buy travel health insurance in this study.
Response:** We appreciate your recommendation. And we’ve revised the sentence according to your recommendation. (LINE 217-221)

**13. 239 – 240 – You should clarify this sentence. According to what you described in 238-239, this sentence should be the cost of travel health insurance for the traveler or you are describing how much it cost without insurance?
Response:** We apologize for confusing you. The cost is the emergency medical evacuation from a low-income nation. If the insurance coverage includes evacuation, then the traveler can pay less. We’ve revised this paragraph. We emphasized the respondents’ neglect of overseas sickness coverage and 24-hour emergency assistance first. Then, we showed that the fee of emergency medical evacuation is high. (LINE 231-234)

**14. 241 - 243 – I suggest:** **Overseas sickness coverage and medical evacuation are crucial while traveling during the Covid-19 pandemic since local healthcare capacity can be overwhelmed.
Response:** Thanks for your suggestion. We’ve revised according to your recommendation. (LINE 238-239)

**15. Grammar correction**

244 – remove “Besides” and “also”
**Response:** We’ve removed this sentence according to another reviewer’s suggestion.

245 – about --> of
**Response:** We’ve removed this sentence according to another reviewer’s suggestion.

249 – of --> to
**Response:** We’ve revised according to your recommendation. (LINE 240)

255 – toward --> of
**Response:** We’ve removed this sentence according to another reviewer’s suggestion.

256 – remove “is”
**Response:** We’ve removed this sentence according to another reviewer’s suggestion.

257 – one third --> one-third
**Response:** Thanks for your suggestion. We’ve revised the sentence to **“Our study revealed only 31.5%–44.7% of respondents understood the content of travel insurance very well, whereas 9.5%–17.3% expressed their ignorance.”** (LINE 222-223)

**16. 267 - 60% general --> 60% of general; give advice --> advise; insurances --> insurance
Response:** We’ve revised according to your recommendation. “ **Recent studies have shown that approximately 60% of General Practitioners in New Zealand [7] and 39% of travel clinics worldwide [8] usually advise travelers to buy travel insurance.**” (LINE 85-87)

**17. 269 – the travelers --> travelers; insurances --> insurance
Response:** We’ve revised the sentences after the last major revision. **“Recent studies have shown that approximately 60% of General Practitioners in New Zealand [7] and 39% of travel clinics worldwide [8] usually advise travelers to buy travel insurance.**” (LINE 85-87)

**18. 271 – insurances --> insurance; a new --> new
Response:** We’ve removed the sentences after the last major revision.

**19. 272 – airline --> airlines
Response:** Thanks for your suggestion. We’ve removed the sentences after the last major revision.

**20. Conclusion**
**The conclusion should include something about knowledge related to infections, vaccines, and illnesses while traveling.
Response:** We’ve added the sentence, **“Understanding local epidemic and implement personal protective measures is still important.”** in Conclusion paragraph. (LINE 272-273)

**21. 289 – 291 I suggest:** **between a third and half of the travelers understand the content of health insurance, almost all recognize its importance, and more than two-thirds were willing to buy a health insurance when traveling.
Response:** Thanks for your suggestion. We’ve revised the sentence according to your recommendation. (LINE 269-271)

**22. 293 – 294 I suggest:** **Therefore, health professionals should be encouraged to acquire health insurance knowledge and transmitted it on during pre-travel consultation.**
**Response:** We’ve revised the sentence according to your recommendation. (LINE 274-276)

**23. 295 – consultation --> consultations
Response:** We’ve revised the word according to your recommendation. (LINE 276)

**24. Figure 1 must be redone, it is not possible to see due to lack of quality.
Response:** Thanks for your suggestion. We’ve removed Figure 1 according to the last reviewer’s suggestion.

**Reviewer #3:**

**I would like to thank the editors for giving me the opportunity to review the manuscript "Traveler’s Knowledge, Attitude, and Practice about Travel Health Insurance” which was submitted to ‘PLOS ONE'. I found the manuscript intriguing and informative. However, in my opinion, this manuscript cannot be published in its current form, certain corrections should be made. Therefore, based on my observations, I am making the following suggestions to improve the quality of the current work.
1. First and foremost, I'd like to thank to all of the authors for their strong effort. However, I found the manuscript's 'research gap and research objective' is not clear. The research gap should be clearly mentioned in the introduction part so that we can get a clear idea. For example, the following statements in the 'introduction section' is not clear and should be rewritten.
Introduction (lines no. 96 to 99):
'In addition, the travelers' awareness---------have not been well investigated'.**
**Response**: Thanks for your comments. We’ve revised the paragraph of introduction according to your suggestion. (LINE 92-99)

**2. It is unclear Why the authors collected data on "willingness toward visiting travel medicine clinics". They emphasized on "travel insurance purchase behavior" rather than "willingness travel medicine clinics visit" in their research objectives. This should be included in your research objective or left out of the manuscript.
Response**: Thanks for your comments. Pretravel consultation is also important in addition to buying travel health insurance. In fact, pretravel consultation is our prime opportunity to educate travelers. Thus, we want to understand the correlation to clarify what else we could do. So, we include the willingness to seek pre-travel consultation in our research objective. (LINE 94-95)

Questionnaire section (line no 119)
**3. The authors mentioned about the pretest of the questionnaires, in the questionnaire section, which is commendable. However, the measurement scales of (lines 129 to 147) 'knowledge', 'attitude' and 'purchase behavior' are well established in the fields of marketing and management. It is unclear why the author did not adapt those scale to conduct the study. Moreover, a pretest alone is insufficient for developing a measurement scale, and it may cause concerns with the scale's validity and reliability. I believe that additional information in this area is required.
Response**: Thanks for your comments. Reviewing the literatures, most studies focus on health insurance rather than travel health insurance [1-3]. Our last study focused on emergency medical assistance services [4]. Because this time we wanted to know travelers’ knowledge, attitude, and purchase behavior of different kinds of travel insurance, we used our questionnaire which designed by ourselves. The Cronbach’s Alpha is 0.927, which represents good reliability. About validity, ten physicians with clinical experienced in travel medicine conducted face validity. So, we believe our questionnaire is qualified.

Reference:
1. Sheryl AR, Margarete E, Charles EI. Adolescents’ knowledge of their health insurance coverage. J Adolesc Health. 1998;22:293-299
2. Dhungana BR, Giri SS, Kushwaha SP, Khanal DK, Yadav BK. Awareness and Perception Regarding Health Insurance Among Community People in a Municipality: A Cross Sectional Study. MedS J Med Sci. 2021;1:76-82.
3. Evita A, Enrico MB, Gian PS. Understanding Insurance Knowledge: A Brief 7-Item Measure. Int J Bus Manag. 2021,16 (2), 65-74.
4. Lee YH, Lu CW, Wu PZ, Huang HL, Wu YC, Huang KC. Attitudes and awareness of medical assistance while traveling abroad. Global Health. 2018;14(1):67. doi:10.1186/s12992-018-0382-5.

**4.The findings of tables 1,2 and 3 should be properly discussed in the results section. The authors just mentioned a proportion of the table 1 and 2 and there was no interpretation for table 3. Please appropriately discuss the tables based on your research objective so that readers comprehend the research findings.**
**Response**: Thanks for your comments. Table 2 is our main research objective, so we wrote more about table 2. We’ve revised the discussion paragraph according to your suggestion. Our interpretation for table 3 is on LINE 240-246. We emphasize travelers who have the willingness to buy travel health insurance may not have good knowledge of travel medicine.

**5. No justification was provided for using one-way ANOVA and the t-test to analyze the data. Please provide justifications for using these statistical analyses to analyze the research data. Besides, the authors mentioned 'socio-demographic" variables in line 193 and 'socio-economic" variables inline 195 (under table 3). The terminology inconsistency should be addressed.
Response**: Thanks for your recommendation. We’ve revised the terminology inconsistency. There were 10 questions to test travelers’ knowledge of travel medicine. Each question was scored 1 point for a correct response, with a total score of 10 points, so it was a continuous variable. The socio-demographic variables included age, medical history, travel-associated illness and planned special activities during travel. We divided the respondents into 2 and 5 groups, so we used one-way analysis of variance (5 groups) and independent t-test (2) groups to clarify the relation between the knowledge of travel medicine and socio-demographic variables.

**6. The data analysis from the discussion section can be transferred to the result section. It is better if the authors compare their findings to previous studies in this field and provide an explanation for any discrepancies in their findings based on prior research and theory. In the discussion section, they should also include the research contribution, which should address the research gap and objectives. Please revise the "Result" and "Discussion" sections.
Response**: Thanks for your recommendation. We’ve revised the Result and Discussion sections according to your suggestion.

**6. It is mentioned that travelers rely more on "healthcare workers" (lines 241 to 246). However, the information about healthcare workers was not provided in any table.
Response:** We’ve added Table 4 to show the source and subjective trust of travel health insurance information.

**7. In the limitation section, the authors mentioned about non-response bias. This I believe is not a problem because 92.7% responses are already good enough. However, I am concerned that the authors stated that they were unable to demonstrate a causal relationship due to the cross-sectional study (lines 254 to 256) ! Cross-sectional studies are commonly used in social science studies to examine the causal relationship. There are numerous ways to addressing the bias of cross-sectional studies (e.g. marker variables). If the authors want, I believe they can use "Structural Equation Modeling" to provide greater understanding into the phenomenon. It is very important to understand the research objectives and what methodologies we are going to adopt to conduct the research.
Response**: Thanks for your recommendation. We agreed! We’ve revised the Limitations section. This is the pilot study to understand the traveler’s knowledge, attitude, and practice about travel health insurance. We’ll analyze the factors which influence their behavior in the future.

Reviewer #4:

**The authors have addressed the reviewer's comments satisfactorily. An additional point should be mentioned in the discussion. How do the authors propose that travel medicine professionals educate themselves about the various types of travel insurance policies? This topic is rarely the exclusive topic of any conference session, although it is mentioned a lot in passing. Insurance medicine is itself a branch of medicine. Should there be greater cooperation between the two disciplines perhaps? Should we invite insurance physicians to attend our conferences or present at our webinars? A final point relates to the references. The authors correctly state that there has been limited previous work in this area. They may have overlooked the following article which is available as an open access source. There may be useful material that could strengthen your discussion. Source:** **Darrat M, Flaherty GT. An Exploratory Study of Medical Cover Policies Offered by the Travel Health Insurance Industry. International Journal of Medical Research & Health Sciences, 2019, 8(8): 1-8**

**Response**: We appreciate your recommendation. If there are more cooperation between the two disciplines, we believe that travelers can receive more comprehensive care. (LINE 219-221) We have also added this study finding into our discussion section. (LINE 224-226, 254-255) (Darrat M, Flaherty GT. An Exploratory Study of Medical Cover Policies Offered by the Travel Health Insurance Industry. International Journal of Medical Research & Health Sciences, 2019, 8(8): 1-8)
